# The clinical spectrum associated with ERCC5 mutations: Is there a relationship between phenotype and genotype?

**DOI:** 10.1002/pdi3.71

**Published:** 2024-07-01

**Authors:** Jinpeng Zhang, Jiannan Ma, Yuanyuan Luo, Siqi Hong, Li Jiang, Tianyi Li

**Affiliations:** ^1^ Department of Infection Children's Hospital of Chongqing Medical University National Clinical Research Center for Child Health and Disorders Ministry of Education Key Laboratory of Child Development and Disorders, Chongqing Key Laboratory of Child Infection and Immunity Chongqing China; ^2^ Department of Neurology Children's Hospital of Chongqing Medical University National Clinical Research Center for Child Health and Disorders Ministry of Education Key Laboratory of Child Development and Disorders Chongqing Key Laboratory of Pediatrics Chongqing China

**Keywords:** Cockayne syndrome, COFS, ERCC5, liver function injury, Xeroderma pigmentosum

## Abstract

Mutations in the *ERCC5* gene can lead to different clinical phenotypes, few articles have reported the clinical phenotypes in detail and explained the relationship between genotype and phenotype. The clinical data of cases with *ERCC5* gene mutations diagnosed in our center and reported in previous studies were collected. The cases were divided into three groups based on phenotype; the differences of clinical manifestation and genotype among groups were analyzed. Genetic tests showed a complex heterozygous mutation of the *ERCC5* gene with paternal C.402_C.403 (exon 4) insA (p.T135Nfs*28) and maternal C.1096 (exon 8) C > T (p.R366X.821) in our case. The gene mutation has not been reported and was predicted to seriously affect the protein structure. According to a review of 59 cases of *ERCC5* mutations, cerebrooculofacioskeletal syndrome (COFS) occurred in 16 cases, XP in 19 cases, and XP/CS in 24 cases. The incidence of physical retardation, mental retardation, peripheral neuropathy, magnetic resonance abnormalities and fundus/vision abnormalities in XP/CS patients was significantly higher than that in XP patients. In addition, patients with the XP/CS phenotype were more prone to appearance abnormalities, deafness, and epilepsy, and cheilitis and tumors were more common in patients with the XP phenotype, but the differences were not significant. XP/CS can cause abnormal liver function and even fatality, which should be given attention. *ERCC5* mutation‐related diseases were characterized by mild to severe clinical phenotypes. In addition to tumors, liver function should be considered in *ERCC5*‐related diseases, and patients should be cautious with medication to avoid drug‐induced liver injury.

## Graphical abstract

The clinical manifestations of the syndrome including photophobia, photosensitive rash, sclera jaundice, freckle‐like pigmentation and so on caused by ERCC5 mutation. Liver function damage in XP/CS phenotype is like snowflakes in the cold winter.

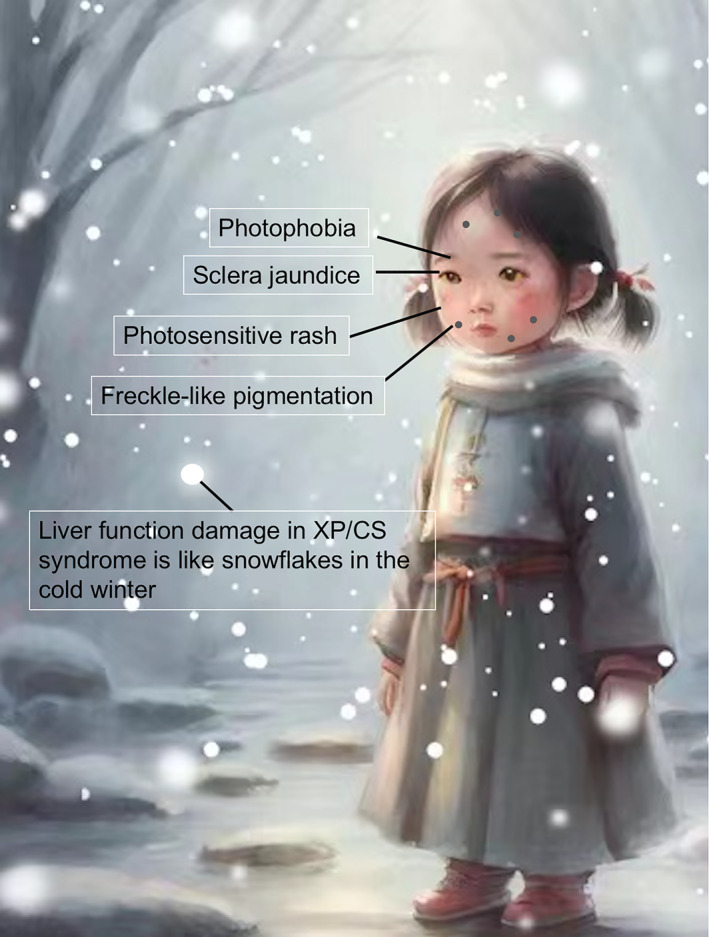

## 1 BACKGROUND

Xeroderma pigmentosum (XP) is a group of rare autosomal recessive genetic diseases. The main pathogenesis of XP is the impairment of DNA damage repair ability caused by some gene mutations, which leads to photosensitivity, skin damage, ocular lesions, nerve damage, malignant tumors, developmental disorders, and other clinical manifestations. These clinical manifestations are also the phenotypes of classic XP. There are seven complementary types (XPA, XPB, XPC, XPD, XPE, XPF, and XPG) and one variant type (XPV) of XP, corresponding to eight gene mutations (*XPA*, *ERCC3*, *XPC*, *ERCC2*, *DDB2*, *ERCC4*, *ERCC5*, and *POLH*).^1^, ^2^ XPA has a hot spot gene mutation site, c.555 + 8A>G, which results in no neurological abnormalities and fewer eye diseases but is prone to skin cancer.^3^ Other gene mutations can lead to developmental delay, microcephaly, and other neurodevelopmental abnormalities. The number of patients with the XPB type is rare, and they exhibit photosensitivity, but except for sensory deafness, neurological dysfunction is not obvious.^4^ XPC patients are particularly sensitive to ocular injury, and ocular disease is more serious; most patients do not have progressive nervous system dysfunction, but some patients may develop nervous system tumors.^5^ The hot spot mutation of XPD is c.2047C>T, leading to Arg683TPr.^6^ The light sensitivity of the skin of XPD patients is prominent, and most of them have severe and lasting sunburn by the age of 1 year. XPE patients usually do not have any obvious neurological abnormalities, but they have a high incidence of skin cancer, with more than 100 basal cell carcinomas and squamous cell carcinomas.^7^ Some patients with XPF have severe sunburn in the early stage, but they do not develop skin manifestations after young adulthood.^8^ Patients with XPV have basically normal neurodevelopment and develop multiple skin cancers between the ages of 20 and 30.^1^ According to previous reports, XPG patients have a wide range of clinical phenotypes, from XP to severe XP/CS phenotypes and even cerebrooculofacioskeletal syndrome (COFS).

With an increasing number of XP patients diagnosed worldwide, some XP patients have been found to present with peripheral sensory neuropathy, progressive cerebellar ataxia, dysarthria, sensorineural hearing loss, progressive cognitive decline, depressed eyes and subcutaneous fat loss. These features are consistent with late‐onset Cockayne syndrome (CS), and this phenotype is defined as XP complicated with CS (XP/CS).^9^, ^10^ However, some patients are more severe and present with COFS, which is characterized by fatal growth failure and progressive neurodevelopmental abnormalities in infancy.^11^

However, our understanding of the clinical manifestations of XPG is limited, and the relationship between genotype and phenotype is controversial. To better understand XPG, we reported a case of XP/CS caused by de novo compound heterozygous mutations of *ERCC5*. The clinical phenotypes and gene mutations of patients with *ERCC5* mutations reported in the literature were analyzed.

## 2 METHODS

We reported a group of children with an XP/CS phenotype caused by a new mutation of *ERCC5* diagnosed in Children's Hospital of Chongqing Medical University, and the clinical data including general information, appearance abnormalities, skin abnormalities, neurological symptoms, auxiliary examination results, mutated forms, and other information of cases with *ERCC5* gene mutations reported in previous studies were collected. The cases were divided into three groups based on phenotype: XP, XP/CS, and COFS. The differences of clinical manifestation and genotype between groups were analyzed. SPSS 20.0 statistical software was used for statistical analysis; statistical methods include *t* test, chi‐square test, and one‐way analysis of variance.

## 3 RESULTS

A 4‐year‐old girl complained of developmental delay with recurrent rash for 3 years and jaundice of the skin and sclera for half a month. At the age of 1 year, erythema and swelling gradually appeared on the head, face, and hands after sun exposure, followed by rough and dry skin. A freckle‐like rash and pigmented nevus mainly appeared on the exposed site. She had delayed motor and intellectual development since childhood. She raised her head at the age of 1 year, sat alone at the age of 6 months and was unable to walk alone until recently. She can understand simple instructions but thus far cannot call mom and dad. Her parents were not consanguineous, and there was no family history of similar diseases. The mother had two induced abortions, and G1P1 is a healthy 12‐year‐old girl. Half a month prior, the patient developed jaundiced skin and sclera due to cough and runny nose.

**FIGURE 1:**
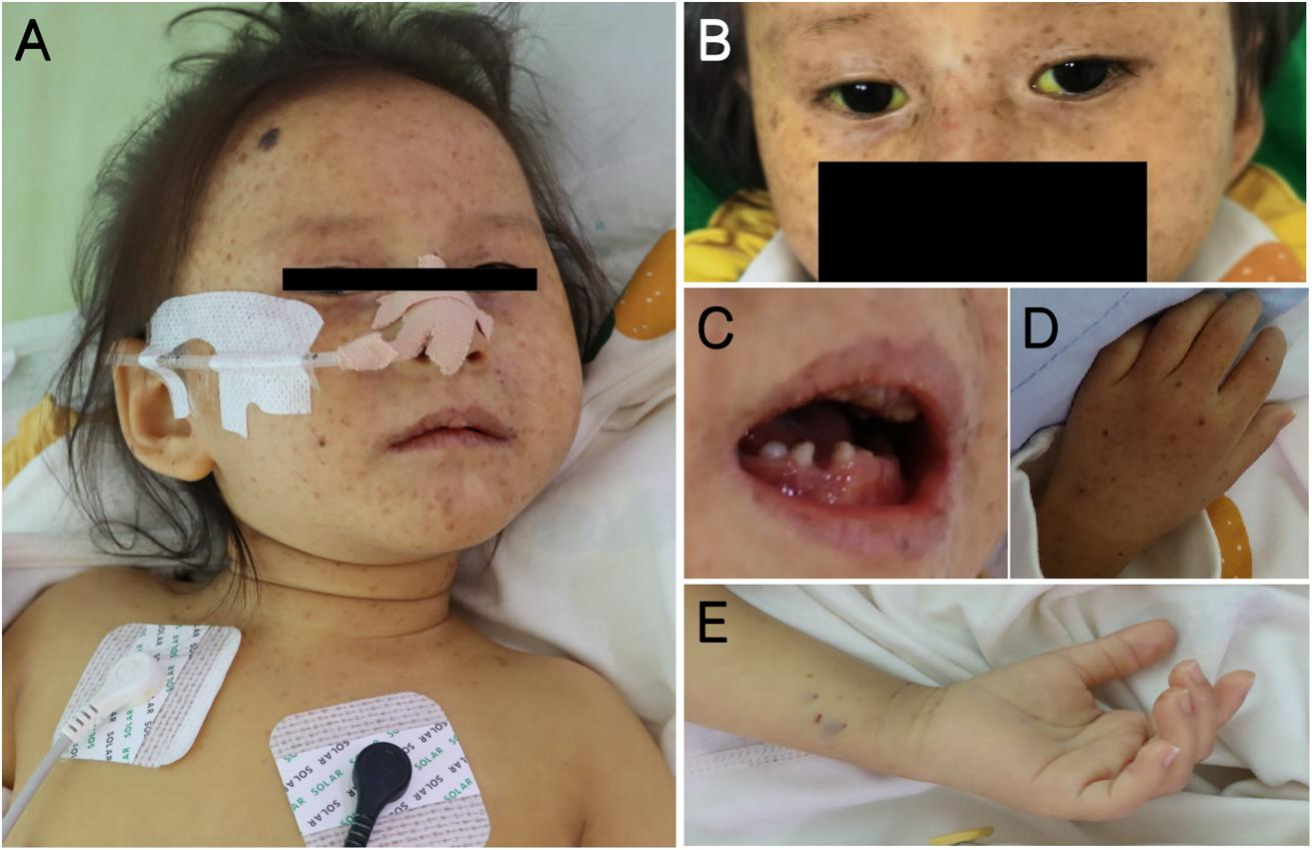
The clinical manifestations of our case. Freckle‐like pigmentation can be seen on the face and hands, and there are also slightly larger pigmented moles (A, B, D), There was no obvious abnormality in the skin on the flexion side of the arm (E). The sclera showed obvious jaundice (B). Dental dysplasia was present in this case (C).

Physical examination showed stable vital signs, weight of 8 kg, length of 82 cm, and head circumference of 40 cm. She exhibited photophobia to the point of covering her eyes with a quilt. The patient's teeth were sparse, uneven, and underdeveloped. There were patchy brown rashes on the face, ears, neck, and back of both hands, interspersed with hypopigmentary spots. She exhibited jaundiced skin of the face, whole body, and sclera (Figure 1). Cardiopulmonary examination revealed no abnormalities. The abdomen was soft, and the liver measured 1.5 cm subcostal and 3 cm subxiphoid and was soft with sharp margins and no tenderness. The spleen was not palpable under the ribs, and there was no crying, rebound or muscle tension during whole abdominal compression.

Nervous system examination showed clear consciousness; involuntary jitter and tremor of the eyes, limbs, and head; uncooperative muscle strength examination of the limbs; increased muscle tone of both lower limbs; hyperreflexia of the knee tendon; and positive ankle jerk. Tests for Kirschner's disease, Brucellosis, and Bartter's disease were negative.

### 3.1 Auxiliary examination

Biochemical tests showed the following results: alanine aminotransferase, 73 U/L; aspartate aminotransferase, 167 U/L; total bilirubin, 260.3 μmoL/L; conjugated bilirubin, 192.05 μmoL/L; albumin, 26 g/L; and bile acid, 28 μmoL/L. Routine blood tests showed normal white blood cell count (11*10^9/L) and hemoglobin level (74 g/L). Test for EB virus, cytomegalovirus, respiratory syncytial virus, influenza, mycoplasma, and other respiratory pathogens were negative. The hepatitis markers CRP and PCT were negative. Magnetic resonance cholangiopancreatography showed no biliary mass, atresia, or inflammation.

**FIGURE 2:**
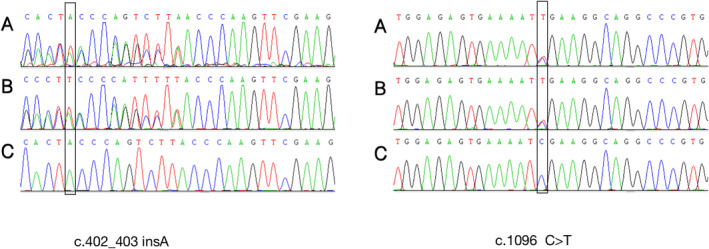
The mutation sites of the patient in our hospital, (A) the patient, (B) the father, and (C) the mother.

The Wechsler intelligence scale score was 45. No sensorineural deafness was found by pure tone audiometry. Brain magnetic resonance imaging showed symmetrical abnormal signal shadows in the bilateral lenticular nucleus. Bilateral frontotemporal, parietal, and occipital subdural effusion with slight widening of the left pontocerebellum were observed (Figure 2). Chest and abdominal CT scans showed hepatomegaly, and no lymph node enlargement or space‐occupying lesions were found.

The patient was treated with compound glycyrrhizin, ademetionine, red blood cell transfusion, albumin transfusion, blood purification, and other treatments. The jaundice of the skin gradually worsened. She was discharged from the hospital and died 2 weeks later. No autopsy was performed.

**FIGURE 3:**
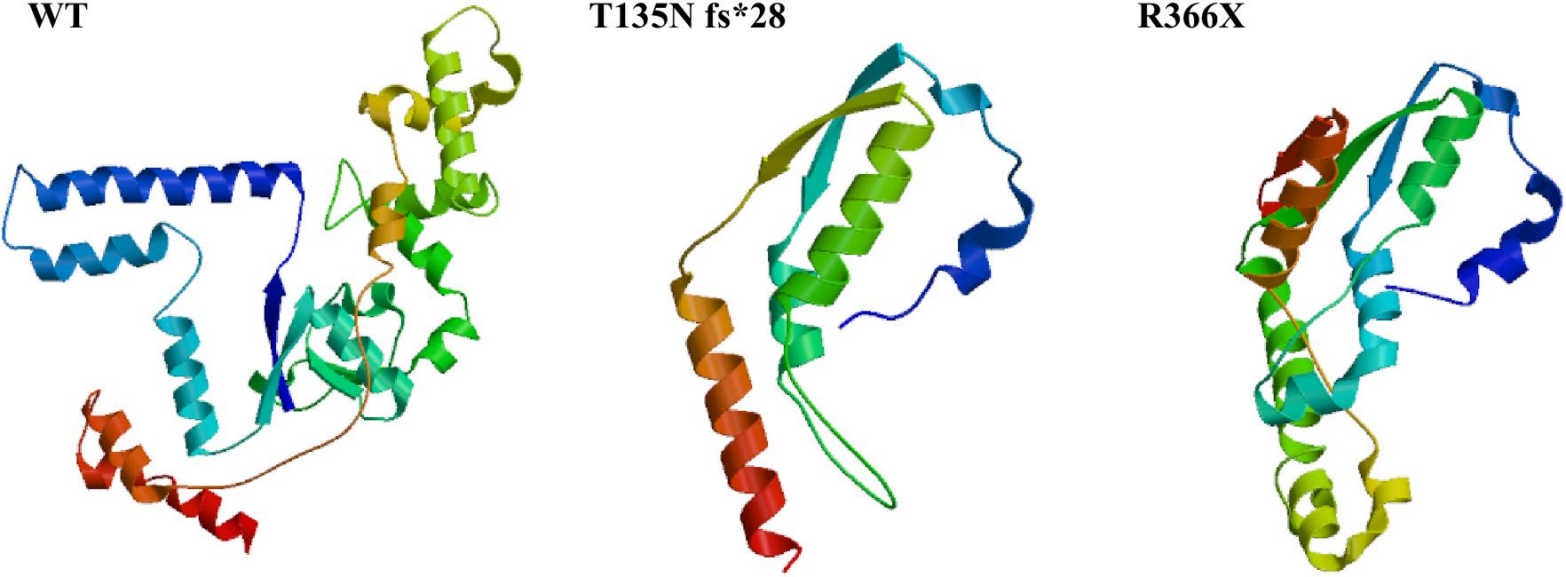
Protein structure prediction can be seen that mutations lead to significant structural changes, which may affect protein function.

**FIGURE 4:**
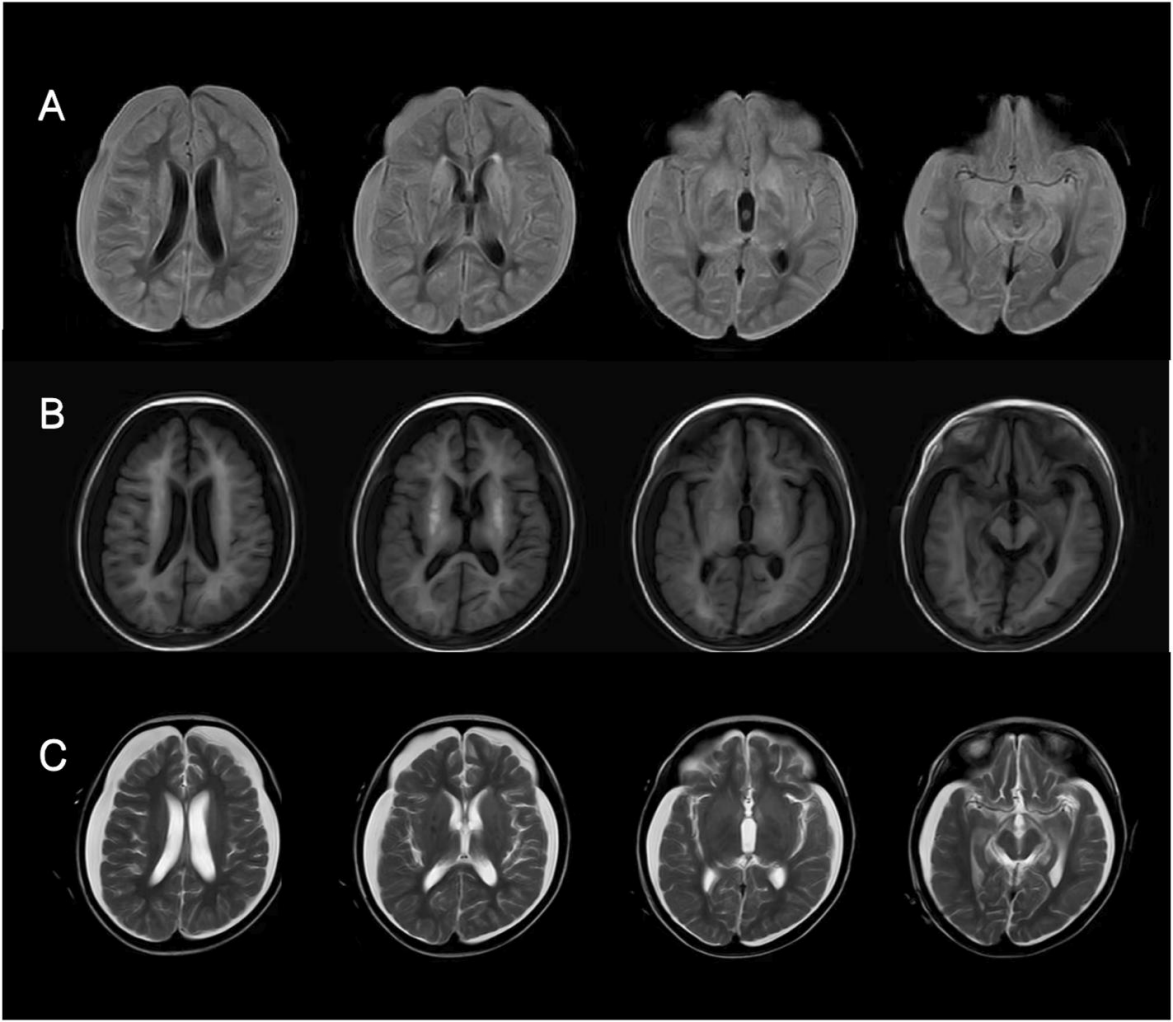
Brain MRI images of the patient in our hospital, (A) flair, (B) T1, and (C) T2.

Whole‐exome sequencing (WES) and Sanger sequencing verification showed compound heterozygous mutations of the ERCC5 gene, C.402_C.403 (exon4) insA (p.T135Nfs *28), and C.1096 (exon 8) C > T (p.R366X.821) (Figure 3). Neither mutation had been reported before; were not found or had a very low frequency in the Genomes, GnomAD, TOPMED, and other healthy population control databases; and were not polymorphic sites. Protein structure prediction showed significant changes in protein functional structure (Figure 4).

### 3.2 Literature review

By reviewing the literature, 59 previously reported children with ERCC5 mutations were included and grouped according to phenotype, including 16 cases of COFS, 19 cases of XP, and 24 cases of XP/CS (Tables 1, 2, 3).

**TABLE 1:** Clinical data of patients of XP phenotype caused by *ERCC5* mutation.

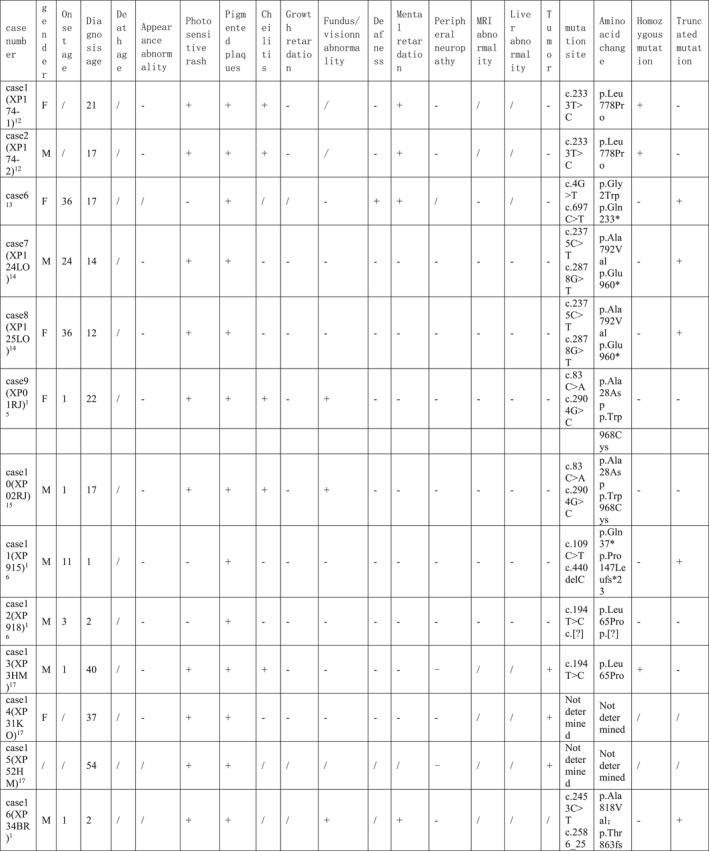
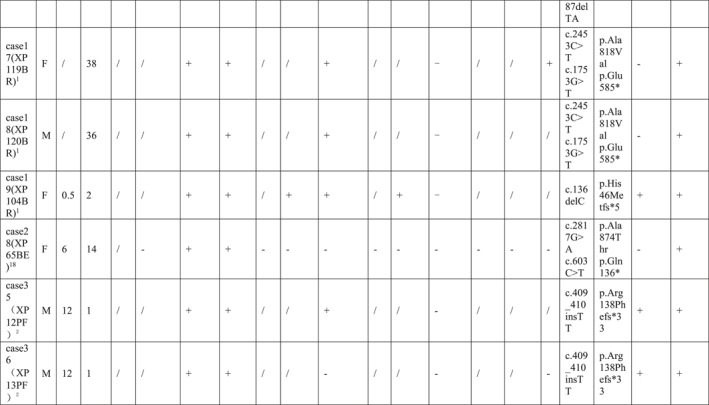

**TABLE 2:** Clinical data of patients of XP/CS phenotype caused by *ERCC5* mutation.

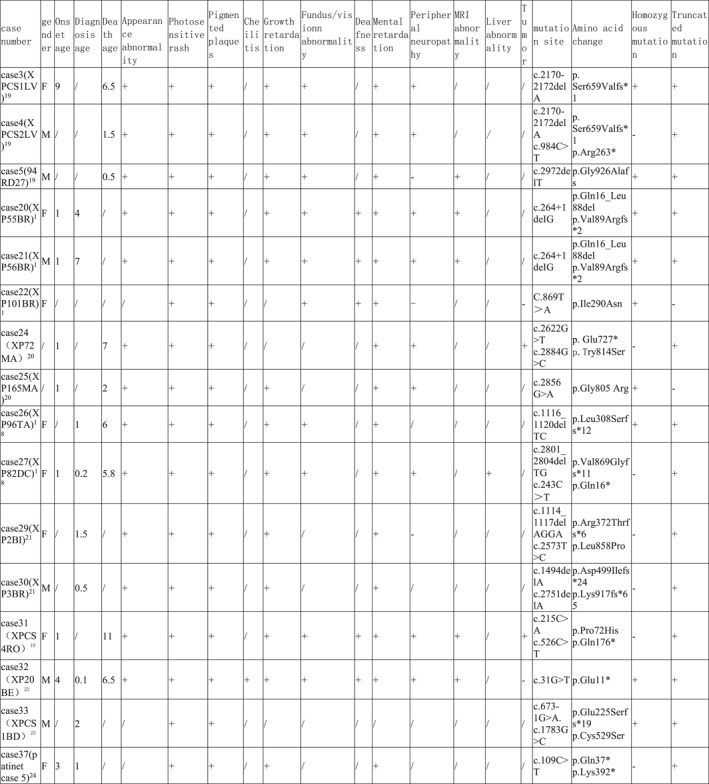
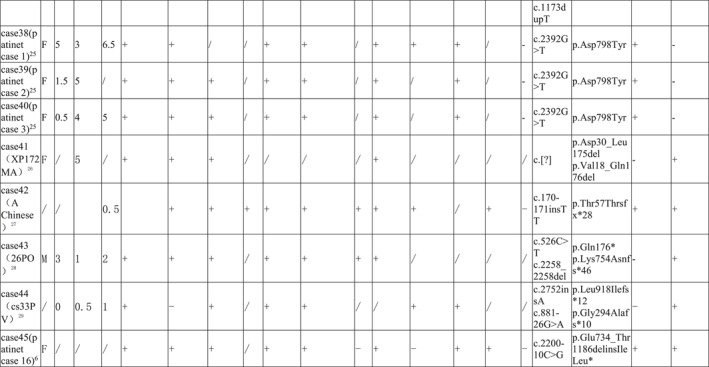

**TABLE 3:** Clinical data and statistical analysis of XP, XP/CS, and COFS phenotypes caused by *ERCC5* mutation.

Phenotype		XP‐G	XP/CS	COFS^11^, ^30^, ^31^, ^32^, ^33^
Number		19	24	16
Gender (male: female)		10: 8	7: 13	/
Onset age M, month		11.1	2.3*	/
Diagnosis age M, year		18.3	2.4***	/
Dead *n*, %		/	14 (58.3%)****	16 (100%)
Death age M, year		/	4.4	TOP‐4w
Appearance abnormality	Microcephaly *n*, %	/	12 (50%)***	/
	Sunken‐eyed *n*, %	/	4 (16.7%)	/
	Limb abnormalities *n*, %	/	5 (20.8%)	/
	Micrognathia *n*, %	/	3 (12.5%)	/
	Lowset ears *n*, %	/	2 (8.3%)	/
	Micropenis *n*, %	/	1 (4.2%)	/
	Cleft palate *n*, %	/	1 (4.2%)	/
	Auricular dysplasia *n*, %	/	1 (4.2%)	/
	Tooth dysplasia *n*, %	/	1 (4.2%)	/
Skin abnormalities	Photosensitive rash *n*, %	16 (84.2%)	23 (95.8%)	/
	Pigmented plaques *n*, %	19 (100%)	23 (95.8%)	/
	Cheilitis *n*, %	3 (15.8%)	2 (8.3%)	/
	Tumor *n*, %	4 (21.1%)	2 (8.3%)	/
Growth retardation *n*, %		1 (5.3%)	20 (83.3%)****	16 (100%)
Fundus/vision abnormality, %		7 (36.8%)	17 (70.8%)*	5 (31.2%)
Deafness *n*, %		1 (5.3%)	7 (29.2%)	/
Mental retardation *n*, %		5 (26.3%)	22 (91.7%)****	/
Peripheral neuropathy *n*, %		/	12 (50%)***	/
Epilepsy *n*, %		/	3 (12.5%)	/
MRI abnormality *n*, %		/	10 (41.7%)**	/
Liver abnormality *n*, %		/	3 (12.5%)	/
Homozygous mutation *n*, %		6 (31.6%)	14 (58.3%)	12 (75%)*
Truncating mutation *n*, %		11 (57.9%)	19 (79.2%)	16 (100%)**

Abbreviation: TOP, termination of pregnancy.

*P < 0.05, **P < 0.005, ***P < 0.0005, ****P < 0.0001.

COFS patients had a poor prognosis, and all died early after birth (16/16, 100%), ranging from immediately after delivery to 4 weeks after birth. All patients with this phenotype had intrauterine growth retardation, microcephaly, and skeletal joint dysplasia (16/16, 100%), and some patients had ocular dysplasia (5/16, 31.2%). Most patients were diagnosed by genetic testing. All mutations in COFS patients were truncating mutations, and the homozygous mutation rate was high. The incidence of truncating mutations and homozygous mutations was higher than that in the XP phenotype (Table 3).

Most patients with the XP phenotype could survive for a long time, but the mortality rate of XP/CS patients was higher than that of XP patients, approximately 58.3%, and the average age of death was 4.4 years. The two phenotypes share the main clinical manifestations of photosensitive rash and hyperpigmentation of light‐exposed areas, while XP/CS patients had an earlier age of onset and diagnosis. Moreover, XP/CS patients were more likely to have physical retardation, mental retardation, peripheral neuropathy, magnetic resonance abnormalities (mainly basal ganglia calcification and cortex and cerebellum atrophy), and fundus/vision abnormalities (83.3% vs. 5.3%, 91.7% vs. 26.3%, 58.3% vs. 0%, 41.7% vs. 0%, and 70.8% vs. 36.8%, respectively). Peripheral neuropathy included decreased or absent reflexes, muscle hypotonia, and reduced sensation. Unfortunately, only a few patients had undergone nerve conduction testing, which had shown decreased nerve conduction velocity. MRI abnormalities include cortical atrophy, cerebellar dysplasia, calcification, and lateral ventricle enlargement.

XP/CS patients were more likely to have appearance abnormalities (including microcephaly, depressed eyes, limb dysplasia, micrognathia, micropenis, cleft palate, auricle dysplasia, and dental dysplasia), deafness and epilepsy, while cheilitis and tumors were more common in XP patients, but the difference was not statistically significant. Notably, some XP/CS patients had abnormal liver function (12.5%), as in our case. Cases 27, 42, and 45 had abnormal liver function; among them, Case 27 had acute liver failure with suspected viral infection, Case 42 had persistent liver dysfunction, and Case 45 had mild aminotransferase elevation. All four cases with epilepsy were in the XP/CS group. Case 4 had generalized convulsions when he was 8 months old that were controlled by phenobarbital and phenytoin. Case 38 was well controlled by topiramate. Case 31 developed infantile spasms with a burst‐suppression pattern on EEG at the age of 8 months. Case 44 suffered weekly focal motor seizures associated with multifocal epileptic abnormalities from the age of 8 months. Ocular abnormalities included retinal pigmentation, vision loss, and cataracts.

In terms of gene mutation forms, there was no difference in the truncating mutation rate and homozygous mutation rate between XP and XP/CS phenotypes.

## 4 DISCUSSION

XP is a group of diseases caused by defects in the genes of proteins involved in the nucleotide excision repair machinery, including XPA‐XPG types. Nucleotide excision repair (NER) can repair DNA damage caused by ultraviolet rays, chemical reagents, toxic substances, chemotherapy drugs and REDOX processes. The clinical manifestations of different types of XP are different, and the types of XP also vary by region and race. XPA is more common in Japan; in the United States, XPC is predominant, followed by XPD or XPV. In Europe, XPC accounts for the main proportion, followed by XPV. XPA and XPC are dominant in China.^16^

The XPG phenotype is caused by a defect in the activity of the structure‐specific repair endonuclease XPG caused by mutations in the *ERCC5* gene. More than 50 *ERCC5* gene mutations have been associated with the disease. XPG patients present with acute sunburn reaction and freckle pigmentation in the early postnatal period. Some of the combined neurological abnormalities manifested as XP/CS or severe brain‐oculofacial‐skeletal syndrome, and the phenotype was highly heterogeneous.^34^

Through a literature review, it was found that the mutations in COFS patients were all truncating mutations, and the proportion of homozygous mutations was high. However, there was no significant difference in the truncating mutation rate or homozygous mutation rate between the XP group and XP/CS group. The incidences of physical retardation, mental retardation, peripheral neuropathy, magnetic resonance abnormalities, and fundus/vision abnormalities in the XP/CS group were significantly higher than those in the XP group. In addition, XP/CS patients were more likely to have appearance abnormalities (including microcephaly, depressed eyes, limb dysplasia, micrognathia, micropenis, cleft palate, auricle dysplasia, and dental dysplasia), deafness, and epilepsy. Cheilitis and tumors were more common in XP patients, although these differences were not significant.

In addition to the typical manifestations of XP/CS, the patient with the XP/CS phenotype caused by a novel ERCC5 mutation reported in this article developed severe and progressive liver dysfunction after drug induction. Some previously reported XP/CS patients also had liver damage, suggesting that the use of drugs that may cause liver damage should be strictly controlled for patients with the XP/CS phenotype. The analysis of the mechanism may be that nucleotide excision repair proteins are involved in the repair of damage produced by reactive oxygen species, resulting in the loss of cytochrome P450 function, which affects the metabolic function of drugs in the liver and is more likely to cause drug‐ or virus‐induced hepatitis. Moreover, excision repair cross‐complementing (ERCC) genes and key components of the nucleotide excision repair pathway are regarded as crucial factors for DNA repair capacity, and *ERCC5* regulates DNA excision repair, which may contribute to liver function and even cancer risk. In addition, *ERCC5* gene polymorphisms (rs2016073, rs751402, rs2094258, rs2296147, and rs2296148) were found to be associated with cirrhosis and liver cancer.^35^ Some studies have suggested that the *ERCC5* gene polymorphism can be used as a de novo marker of liver disease.^36^ These results indicate that *ERCC5* plays a role in liver metabolism.

## 5 CONCLUSION

Are XP, XP/CS, and COFS different phenotypes caused by the same gene, or are they a spectrum of different severity of the same disease? There is no definitive answer. Our paper presents a good picture of the clinical manifestations of *ERCC5* mutations, which can help clinicians increase the likelihood of earlier identification of this genetic disease. In addition, we also call attention to liver function in this disease. In addition to avoiding light and tumor screening, avoiding the use of drugs that may damage the liver can better protect patients with this disease.

## AUTHOR CONTRIBUTIONS

Tianyi Li designed this research and contributed to writing. Jiannan Ma, Yuanyuan Luo, Siqi Hong, Li Jiang conducted the literature review. Jinpeng Zhang conducted the statistical analysis and contributed to writing. All authors approved the final manuscript.

## CONFLICT OF INTEREST STATEMENT

The authors declare that they have no competing interests.

## ETHICS STATEMENT

The study was approved by the Institutional Ethical Committee of Children's Hospital of Chongqing Medical University and the guardians gave written informed consent prior to obtain the data. The approval number of ethics review is No. 2024‐221.

## CONSENT FOR PUBLICATION

Written informed consent was obtained from the guardians for publication of this study and any accompanying images.

## Data Availability

The data that support the findings of this study are available from the corresponding author upon reasonable request.
